# Condom Negotiation, HIV Testing, and HIV Risks among Women from Alcohol Serving Venues in Cape Town, South Africa

**DOI:** 10.1371/journal.pone.0045631

**Published:** 2012-10-02

**Authors:** Eileen V. Pitpitan, Seth C. Kalichman, Demetria Cain, Lisa A. Eaton, Kate B. Carey, Michael P. Carey, Ofer Harel, Leickness C. Simbayi, Vuyelwa Mehlomakhulu, Kelvin Mwaba

**Affiliations:** 1 Center for Health, Intervention, and Prevention, University of Connecticut, Storrs, Connecticut, United States of America; 2 Department of Behavioral and Social Sciences, Brown University, Providence, Rhode Island, United States of America; 3 Department of Psychiatry and Human Behavior, Brown University, Providence, Rhode Island, United States of America; 4 Centers for Behavioral and Preventive Medicine, The Miriam Hospital, Providence, Rhode Island, United States of America; 5 Human Sciences Research Council, Cape Town, South Africa; 6 University of the Western Cape, Cape Town, South Africa; Yale School of Public Health, United States of America

## Abstract

**Background:**

Women in South Africa are at particularly high-risk for HIV infection and are dependent on their male partners' use of condoms for sexual risk reduction. However, many women are afraid to discuss condoms with male partners, placing them at higher risk of HIV infection.

**Purpose:**

To examine the association between fear of condom negotiation with HIV testing and transmission risk behaviors, including alcohol use and sexual risks among South African women.

**Method:**

Women (N = 1333) residing in a primarily Xhosa-speaking African township in Cape Town and attending informal alcohol-serving venues (shebeens) completed anonymous surveys. Logistic regression was used to test the hypothesis that fear of condom negotiation would be associated with increased risk for HIV.

**Results:**

Compared to women who did not fear condom negotiation, those who did were significantly less likely to have been tested for HIV, were more likely to have experienced relationship abuse, and to report more alcohol use and more unprotected sex.

**Conclusions:**

For women in South Africa, fear of condom negotiation is related to higher risk of HIV. HIV prevention efforts, including targeted HIV counseling and testing, must directly address gender issues.

## Introduction

South Africa has the largest number of people living with HIV/AIDS with an estimated 5.6 million people [1]. Women are particularly vulnerable; in 2008, HIV prevalence was 20% among women and 12% among men aged 15–49 years [2]. Women in South Africa are also in a position of low status and power relative to men in economic, political, and social arenas. This gender-based power imbalance has been shown to place women at an increased vulnerability to HIV infection, driven particularly by the experience of gender-based violence [3], [4]. For example, research has demonstrated how gender-based violence perpetrated by men against women can increase the latter's vulnerability to HIV infection. This increase occurs as a result of factors like alcohol use and unprotected sex [5]. Experiencing violence from a partner may also have a more generalized impact on HIV prevention behaviors. Specifically, women who have been physically and/or sexually abused report more difficulty discussing condoms with their partners; this fear of discussing condoms with sex partners places women at high-risk for HIV infection.

Despite relatively high levels of knowledge about HIV and AIDS, condom use remains inconsistent among Southern African women and men [6]–[8]. Indeed, maintaining a relationship can often take precedence over health concerns, particularly among women who are dependent on men for their economic resources. Thus, despite knowing about HIV and perhaps even having general discussions with others about the issue, women may still avoid negotiating condoms with their partner [9], [10]. These contextual factors surrounding women's fear of condom negotiation help provide context to understanding women's risks for HIV, and need to be included in gender-specific HIV prevention interventions. It is also possible that gender-related barriers to HIV/STI preventive behavior may extend beyond condom use to impede HIV risk reduction more generally. For example, women's fear of condom negotiation may also be associated with reluctance to seek HIV testing.

Women who are afraid of discussing condoms with their partners may also be fearful of discovering their HIV status. Given the stigma attached to HIV/AIDS, people are often reluctant to seek HIV testing [11], [12]. Indeed, research has shown that the benefits of HIV testing and early HIV treatment may be outweighed by the negative impact of learning one's HIV status [13]. However, given women's higher risk for HIV infection, it is imperative that those women who fear discussing condoms get tested for HIV in order to begin antiretroviral therapy and avoid infecting others. To our knowledge, however, no previous study has been conducted to examine whether fear of condom negotiation may be a barrier to HIV testing among women in South Africa.

Finally, women's fear of discussing condoms may not only be related to sexual risks and HIV testing, it may also be associated with alcohol-related risk. In Sub-Saharan Africa, alcohol use is robustly associated with high-risk sexual behavior [14], [15]. In South Africa, beyond alcohol impairing decision-making, drinking environments promote elevated rates of alcohol use and sexual risk behavior [15], [16]. People often drink in informal alcohol-serving environments called shebeens, and it is common to meet sex partners in these venues [16]. To our knowledge, previous research has not examined fear of condom negotiation among women who patronize shebeens in South Africa.

In the present study, we examine fear of condom negotiation among women in a township in Cape Town, South Africa. We compared women who report fear of discussing condoms with a partner to women who do not in regards to HIV testing, alcohol use, rates of sexual behaviors, and unprotected sex. We also examined contextual variables surrounding fear of condom negotiation, including relationship abuse and discussing HIV/AIDS and condoms with others. We hypothesized that women who feared discussing condoms with a partner were more likely to have experienced abuse from a partner, were more likely to report higher alcohol use, sexual risk behaviors, unprotected sex, and were less likely to report being HIV tested.

## Methods

### Participants

A total of 2367 women residing in a primarily Xhosa-speaking African township just outside Cape Town, South Africa were surveyed. All participants were age 18 or older. Nearly all (98%) participants identified as Black African, 51% were unemployed and 50% had not matriculated high school.

### Research Setting and Procedures

The current study was conducted in a township located about 20 kilometers (km) outside of Cape Town's central business district. Residents are primarily of Xhosa cultural heritage. Neighborhoods were defined as areas approximately ½ km wide that contained at least one informal drinking venue (i.e., shebeen). We used methods described by Weir et al. to perform community assessments to identify 10 shebeens separated by at least 1 km from each other [17]. All shebeens were confirmed by physical visits when we interviewed owners, managers, and patrons to determine whether the shebeens served sufficient numbers of persons to warrant inclusion. We selected shebeens that served at least 75 patrons per week.

Field workers surveyed persons on the street (16%) as well as individuals socializing and drinking in the neighborhood shebeens (84%). Field workers were 8 indigenous men and women from communities similar to our selected township and spoke both Xhosa and English. Men and women were asked if they wanted to complete an anonymous survey to help their community. Participants who agreed (95%) were given a 9-page anonymous survey that most completed within 15–20 minutes. Participants were compensated for taking the time to complete the survey with a keychain or shopping bag. Surveys were collected inside (48% of men and 37% of women) and outside (52% of men and 63% of women) of the shebeens in the target neighborhoods. Surveys were self-administered, printed in either English or Xhosa, and interviewer assistance was provided if needed (<5%). Surveys were not reviewed in the field and no names were collected with surveys to protect participant anonymity.

### Ethics Statement

We obtained verbal informed consent from participants. Written consent was not obtained to ensure participant anonymity. As previously described, participants were approached and asked to completed the anonymous survey. The cover page of the survey described the survey as anonymous, instructed participants to not write their name anywhere on the survey, and provided them with the option of stopping the survey at any time and skipping any questions. Participants were also given a written copy of the information sheet about the study that included information about the researchers and their contact information. Following this procedure, survey completion by the participant served as documentation of verbal informed consent. Institutional Review Boards of the University of Connecticut, the Human Sciences Research Council, and Syracuse University approved all study and consent procedures.

### Measures

Participants were asked to report demographic characteristics, alcohol use, shebeen attendance, HIV risk history, and sexual risk behaviors.

#### Demographic characteristics

Participants reported their age, race, cultural heritage, education, marital status, employment status, and whether they had any children.

#### Fear partner in condom negotiation

Participants were asked when was the last time they “have been afraid to ask a partner to use condoms because he might get angry.” They responded “never,” “in the past 30 days,” or “I have done this, but not in the past 30 days.”

#### HIV-related discussions and relationship violence

Participants were asked about the last time they: “Talked with people in your community about HIV/AIDS;” “Talked to someone about getting tested for HIV;” “Advised someone to use condoms;” and “Were forced to have sex when you did not want to.” Again, response choices were “never,” “in the past 30 days,” and “I have done this, but not in the past 30 days.”

#### Risk associated behaviors

We conceptualized HIV transmission risk as alcohol-related risk, sexual risk behavior, and STI/HIV status. Alcohol use. Frequency of drinking was assessed with two items: (a) how often they drank alcohol in the past month and (b) how often they drank in a public place (i.e., bar, tavern, or shebeen). Binge drinking was measured as number of times in the past month a participant drank 5 or more drinks on one occasion as well as an item asking how often participants drank enough to feel intoxicated. Responses included (a) nearly every day, (b) 3–4 times a week, (c) 1–2 times a week, (d) monthly. All alcohol items referenced a standard drink as a single shot of spirits, 340 ml bottle/glass of beer, 1 bottle of cider, or 1 glass of wine. Sexual risk behaviors. Participants reported the last time they met a sex partner in a shebeen. Responses choices were “never,” “in the past 30 days,” and “I have done this, but not in the past 30 days.” Participants also reported the number of male and female sex partners they had in the past month and the number of specific sex acts in which they engaged (vaginal and anal intercourse with and without condoms). We calculated the percent of intercourse occasions unprotected by condoms (total condom unprotected vaginal plus anal acts divided by total protected plus unprotected vaginal and anal acts). Participants also indicated the number of times they talked with a sex partner about using condoms in the past month. Last, participants reported the number of times in the previous month that they drank alcohol before having sex and the number of times they had a sex partner who drank alcohol before having sex. All risk behavior questions were asked with regard to the past month (30 days) and used open response formats, where participants wrote a number of events in a blank space. We selected a one-month time frame and open response formats to improve recall accuracy and provide unanchored responses [18]. Sexually transmitted infection history, HIV testing, and HIV status. Participants indicated if they had been ever been diagnosed with an STI and whether they had been tested for HIV and if so their most recent test result.

### Data Analyses

We analyzed the data in three stages. First, we conducted descriptive analyses of demographics and fear of condom negotiation. We adjusted for demographic differences in our second stage of analyses. In this stage, we used logistic regression to examine the association between fear of condom negotiation with engaging in HIV-related discussions and relationship violence, alcohol use, sexual risk behaviors, STI history, and HIV testing. Finally, we conducted multivariate logistic regression to examine which of the variables significant in univariate analyses would remain significant. Only those variables that were significant at the *p*<.10 level from univariate analyses were included in the multivariate logistic regression.

## Results

A total of 2367 women completed surveys. Women who reported not having any sex partners (449, 19.0%), 464 (24.2%) married women, 70 (4.8%) women who reported more sex partners than actual sex acts (i.e., logic error), and 50 (3.6%) women who only reported sex with other women were not included. Also excluded was one woman who was an extreme outlier on total intercourse who reported having intercourse 302 times in the previous 30 days. The final sample included 1333 heterosexual, unmarried, sexually active women. Among these women, 1193 (89.5%) did not report being afraid to talk to a sex partner about condoms in the previous 30 days and 140 (10.5%) did report recently having this fear. We examined differences between these women in our analyses.

### Demographics

As displayed in Table 1, women who feared discussing condoms with a sex partner were older, more likely to have children, and reported having more sex partners in the previous 30 days than women who did not fear discussing condoms. We controlled for these differences in our subsequent analyses.

**Table 1:** Demographics by fear of partner in condom negotiation (*n* = 1333).

	Fear Partner in Condom Negotiation				
	Not in past 30 days (*n* = 1193)		Yes, in past 30 days (*n* = 140)		
	M	SD	M	SD	OR (95% CI)
**Age**	28.18	6.73	29.92	8.20	1.03** (1.01, 1.06)
**Total sex partners**	1.47	1.55	2.10	2.17	1.14*** (1.06, 1.24)
					
	**n**	**%**	**n**	**%**	
**Ethnicity**					0.48 (.18, 1.30)
Black	1168	98%	134	96%	
Other	21	2%	5	4%	
**Education**					0.82 (.64, 1.06)
No schooling	9	1%	3	2%	
Grade 3 thru Grade 11	505	42%	67	48%	
Grade 12	521	44%	53	38%	
College or beyond	158	13%	17	12%	
**Employed**	470	39%	63	45%	0.80 (.56, 1.14)
**Have children**	920	77%	122	87%	1.96** (1.17, 3.27)

*Notes:* *p<.05, **p<.01, ***p<.001; Fear partner in condom negotiation (0 = no, 1 = yes).

### HIV-related discussions and relationship violence

Table 2 shows data regarding recent experiences with sexual violence and discussing issues related to HIV. Compared to women who did not report fear of condom negotiation, women who did report this fear were more likely to report being raped by a sex partner (6% vs. 24%) and more likely to report recently talking with someone about getting tested for HIV (32% vs. 54%). There was a marginal effect such that they trended to be more likely to report recently talking with someone about HIV/AIDS (43% vs. 51%). There were no statistically significant differences between the two groups in recently advising someone to use condoms (55% vs. 57%).

**Table 2:** Experiences and alcohol use in past 30 days by fear of partner in condom negotiation (*n* = 1333).

	Fear Partner in Condom Negotiation				
	Not in past 30 days (*n* = 1193)		Yes, in past 30 days (*n* = 140)		
	n	%	n	%	AOR (95% CI)
**Been sexually abused (raped)**	69	6%	33	24%	4.90*** (3.07, 7.82)
**Talked with someone about HIV/AIDS**	514	43%	72	51%	1.42† (.99, 2.03)
**Talked with someone about getting tested for HIV**	377	32%	75	54%	2.53*** (1.76, 3.63)
**Advised someone to use condoms**	657	55%	80	57%	1.07 (.75, 1.54)
	**M**	**SD**	**M**	**SD**	
**Alcohol Frequency**	3.29	2.00	3.53	1.83	1.01 (.92, 1.11)
					
**Alcohol Quantity**	9.37	15.47	12.91	22.08	1.01 (1.00, 1.01)
					
**Binge Drinking Frequency**	2.71	1.85	3.36	1.78	1.16** (1.05, 1.27)
**Most # drinks in one drinking episode**	9.89	13.03	11.10	10.15	1.00 (.99, 1.02)
**Intoxication Frequency**	1.71	1.31	2.24	1.53	1.22*** (1.09, 1.37)
**Alcohol use in drinking venues frequency**	3.01	2.00	3.31	1.85	1.03 (.94, 1.12)

*Notes:* †*p*<.10, *p<.05, **p<.01, ***p<.001; Adjusted for age, children, and total sex partners in past 30 days; Fear partner in condom negotiation (0 = no, 1 = yes); Alcohol Frequency, Binge drinking frequency, Intoxication frequency, and Alcohol use in drinking venues frequency: 1 = never to 7 = nearly every day.

### Alcohol use

Table 2 shows differences between participant groups in regards to alcohol use in the past 30 days. Women who reported fear of condom negotiation reported more frequent binge drinking (M = 3.36, 1.78 vs. M = 2.71, SD = 1.85) and drinking to the point of intoxication (M = 2.24, SD = 1.53 vs. M = 1.71, SD = 1.31) than women who did not have this fear.

### Sexual risk behavior

Table 3 shows differences between women who did and who did not fear their partner in condom negotiation on sexual risk behavior. Women who reported fear of discussing condoms were more likely to report meeting a sex partner in a shebeen than women who did not have this fear (30% vs. 9%). Women who reported recent fear also reported more sexual intercourse (M = 19.47, SD = 26.69 vs. M = 12.71, SD = 10.25), more unprotected intercourse (M = 52.10, SD = 36.40), were more likely to report drinking before sex (M = 4.12, SD = 6.02 vs. M = 2.18, SD = 4.31) and were more likely to report that a partner drank alcohol before sex (M = 6.04, SD = 12.88 vs. M = 3.13, SD = 5.05) than women who did not fear their partner in condom negotiation.

**Table 3:** Sexual risk behavior, STI and HIV status, and HIV testing by fear of partner in condom negotiation (*n* = 1333).

	Fear Partner in Condom Negotiation				
	Not in past 30 days (*n* = 1193)		Yes, in past 30 days (*n* = 140)		
*Sexual Risk Behavior*	n	%	n	%	AOR (95% CI)
Met sex partner in shebeen in past 30 days	106	9%	42	30%	3.86*** (2.53, 5.90)

	M	SD	M	SD	
Total intercourse occasions in past 30 days	12.71	10.25	19.47	26.69	1.03*** (1.01, 1.04)
Percent unprotected intercourse in past 30 days	42.3	40.7	52.10	36.40	1.63* (1.04, 2.54)
Talked with partner about condom use in past 30 days	5.15	10.95	8.13	18.78	1.01† (1.00, 1.02)
Drank before sex in past 30 days	2.18	4.31	4.12	6.04	1.05** (1.01, 1.08)
Partner drank before sex in past 30 days	3.13	5.05	6.04	12.88	1.04** (1.01, 1.07)

*STI/HIV status*	n	%	n	%	
STI	482	40%	76	54%	1.65** (1.15, 2.37)
HIV positive^a^	101	10%	22	21%	1.89* (1.10, 3.24)
*Tested for HIV*	986	83%	106	76%	.65* (.42, 1.00)

*Notes:* †*p*<.10, *p<.05, **p<.01, ***p<.001; Adjusted for age, children, and total sex partners in past 30 days; Fear partner in condom negotiation (0 = no, 1 = yes); ^a^among HIV tested persons; STI = sexually transmitted infection

### STI/HIV history and HIV testing

Table 3 shows that women who reported fear of condom negotiation were more likely to have ever been diagnosed with an STI (54% vs. 40%), report being HIV positive (21% vs. 10%), and were less likely to have ever tested for HIV (76% vs. 83%) than women who did not recently fear discussing condoms.

### Multivariate model

Table 4 shows results of the multivariate logistic regression model. All variables that were significantly associated with fear of partner in condom negotiation in bivariate analyses (at the level *p*<.10) were included in this model. Results showed that compared to women who did not recently fear their partner, women who feared a partner in discussing condoms reported significantly higher odds of meeting a sex partner in a shebeen, talking with someone about getting tested for HIV, being raped by a sex partner, and being HIV positive. Women who recently feared condom negotiation were also less likely to have been HIV tested.

**Table 4:** Multivariate model for fear of negotiating condom use among women (*n* = 1333).

			95% CI	
	AOR	Lower		Upper
Age	1.00	0.97		1.04
Total sex partners	1.04	0.94		1.15
Children	1.43	0.77		2.64
Raped by a sex partner	3.62***	2.11		6.19
Talked with someone about HIV/AIDS	1.18	0.77		1.81
Talked with someone about HIV testing	1.86**	1.21		2.86
Binge drinking frequency	1.07	0.94		1.22
Intoxication frequency	1.01	0.86		1.18
Met a sex partner in a shebeen	2.47***	1.49		4.10
Total intercourse occasions	1.02†	1.00		1.04
Unprotected sex (%)	1.32	0.76		2.31
Talked with partner about using condoms	1.00	0.99		1.02
Drank before sex	1.01	0.95		1.07
Partner drank before sex	0.99	0.94		1.04
Sexually transmitted infection	1.42	0.92		2.17
HIV positive	1.89*	1.04		3.45
HIV test	0.35***	0.19		0.63

*Notes:* †*p*<.10, *p<.05, **p<.01, ***p<.001; Fear partner in condom negotiation (0 = no, 1 = yes)

## Discussion

The current study examined fear of condom negotiation as it relates to HIV transmission risk behavior, relationship abuse, and HIV testing among women in South Africa. We hypothesized and found that women who feared discussing condoms with a partner were more likely to also have recently experienced sexual abuse, exhibited higher alcohol use and sexual risk behavior. Moreover, these women were less likely to have been tested for HIV. Thus, women who attend drinking venues in Cape Town who report fear of condom negotiation not only report inconsistent condom use, consistent with previous research, but they also are less likely to get tested for HIV. These data suggest that experiences with relationship abuse from a male partner may be associated with fear of condom negotiation, which in turn may pose a barrier to HIV testing. Future research should examine a process in which experience of relationship violence predicts fear of condom negotiation, which in turn may predict lower likelihood of HIV testing and higher sexual risk behavior using longitudinal methods.

Previous work among low-income African American women has shown that experiencing violence from a male partner is related to fear of discussing condoms, particularly because the suggestion may create a potentially violent situation [19]. Other work among African American women has shown that even despite high levels of knowledge of sexually transmitted infections, women who report fear of abuse from a partner exhibit inconsistent condom use [20]. A more recent study with primarily African American women attending a STI clinic showed that fear of violent consequences to requests for condom use mediated the relationship between history of intimate partner violence and number of episodes of unprotected sex [21]. In the current study, women who have experienced sexual violence from a male partner in the past month were also more likely to report fear of discussing condoms with a partner in the past month. Our data also showed that women who reported such fear were more likely to report having recent discussions with others about HIV and HIV testing. As previous work has shown, individuals often fear the negative impact of discovering their HIV status, and are thus reluctant to seek HIV testing.

In addition to replicating previous work on relationship violence, condom negotiation, and HIV risk behavior using a sample of women in a high-risk setting in South Africa, the current research demonstrates that fear of condom negotiation is negatively related to HIV testing. It may be that women who are aware of the risks involved with inconsistent condom use may also be fearful of discovering their HIV status. This fear may reflect the social stigma associated with HIV. Research in a similar township in Cape Town, South Africa has shown that individuals who endorse stigmatizing beliefs towards people living with HIV/AIDS are less likely to be tested for HIV [12]. Thus, despite having knowledge of the benefits associated with HIV testing and early treatment, women who fear condom negotiation and report inconsistent condom use may remain reluctant to seek testing for fear of experiencing social stigma. Fear of condom negotiation and fear of discovering one's HIV status may also stem from a more generalized avoidance. Future research should more closely examine this potentially complex relationship, as the link between fear of condom negotiation and HIV testing may likely be best understood as one association nested in other issues surrounding fear of violence from a partner and HIV stigma.

The results of this study should be interpreted in light of its limitations. We relied upon self-report of on sexual behavior and alcohol use. Thus, responses may be biased due to social desirability of these private and socially stigmatized behaviors. Although we assessed the women's discussion of HIV-related issues, we cannot be certain that these discussions fostered accurate knowledge of HIV and AIDS. In addition, our sample was drawn by convenience and cannot be considered representative of all Cape Town shebeens, or of South African women in general.

In conclusion, women in high-risk settings of drinking venues in a Cape Town township who report fear of discussing condoms with a partner exhibit higher HIV transmission risks and a lower likelihood of being tested for HIV. HIV prevention efforts, including HIV counseling and testing campaigns, must acknowledge fear of gender-based violence when attempting to teach women about how to safely and effectively negotiate with partners about the use of male condoms.

## References

[1] UNAIDS. Global Report, *UNAIDS report on the global AIDS epidemic 2010* Available: http://www.unaids.org/globalreport/Global_report.htm. Accessed 2012 Aug 27.

[2] Shisana O, Rehle T, Simbayi LC, Zuma K, Jooste S, et al.. (2009) South African National HIV Prevalence, Incidence, Behavior and Communication Survey. HSRC Press.

[3] Dunkle KL, Jewkes RK, Brown HC, Gray GE, McIntyre JA, et al (2004) Gender-based violence, relationship power, and risk of HIV infection in women attending antenatal clinics in South Africa. Lancet 363: 1415–1421. 15121402 10.1016/S0140-6736(04)16098-4

[4] Jewkes RK, Dunkle K, Nduna M, Shai N (2010) Intimate partner violence, relationship power inequity, and incidence of HIV infection in young women in South Africa: a cohort study. Lancet 376: 41–48. 20557928 10.1016/S0140-6736(10)60548-X

[5] Pitpitan EV, Kalichman SC, Eaton LA, Sikkema KJ, Watt MH, et al (2012) Gender-based violence and HIV sexual risk behavior: alcohol use and mental health problems as mediators among women in drinking venues, Cape Town, South Africa. Social Science & Medicine 75: 1417–1425. 22832324 10.1016/j.socscimed.2012.06.020 PMC3425436

[6] Konde-Lule JK, Berkley SF, Downing R (1989) Knowledge, attitudes and practices concerning AIDS in Ugandans. AIDS 3: 513–518. 2508712 10.1097/00002030-198908000-00005

[7] Lindan C, Allen S, Carael M, Nsengumuremyi F, Van de Parre P, et al (1991) Knowledge, attitudes, and perceived risk of AIDS among urban Rwandan women: relationship to HIV infection and behavior change. *AIDS* 5: 993–1002. 1777178 10.1097/00002030-199108000-00011

[8] Wilson D, Msimanga S, Greenspan R (1988) Knowledge about AIDS and self-reported sexual behaviour among adults in Bulawayo, Zimbabwe. Cent Afr J Med 34: 95–97. 3248306

[9] Welch Cline RJ, Johnson SJ, Freeman KE (1992) Talk among sexual partners about AIDS: interpersonal communication for risk reduction or risk enhancement? Health Communication 4: 39–56.

[10] Michael-Johnson P, Bowen SP (1989) AIDS and communication: A matter of influence. University Pub. Group.

[11] Earnshaw VA, Chaudoir SR (2009) From conceptualizing to measuring HIV stigma: a review of HIV stigma mechanism measures. AIDS Behav 13: 1160–1177. 19636699 10.1007/s10461-009-9593-3 PMC4511707

[12] Pitpitan EV, Kalichman SC, Eaton LA, Cain D, Sikkema JK, et al (2012) Gender-based violence, alcohol use, and sexual risk among female patrons of drinking venues in Cape Town, South Africa. Annals of Behavioral Medicine 43: 362–371. 22526526 10.1007/s10865-012-9423-3 PMC3646073

[13] Jürgensen M, Tuba M, Fylkesnes K, Blystad A (2012) The burden of knowing: balancing benefits and barriers in HIV testing decisions: a qualitative study from Zambia. BMC Health Serv Res. doi: 10.1186/1472-6963-12-2. 10.1186/1472-6963-12-2 PMC3268706 22222028

[14] Kalichman SC, Simbayi LC, Kaufman M, Cain D, Jooste S (2007) Alcohol use and sexual risks for HIV/AIDS in sub-Saharan Africa: systematic review of empirical findings. Prev Sci 8: 141–151. 17265194 10.1007/s11121-006-0061-2

[15] Morojele NK, Kachieng'a MA, Mokoko E, Nkoko E, Parry CDH, et al (2006) Alcohol use and sexual behaviour among risky drinkers and bar and shebeen patrons in Gauteng province, South Africa. Soc Sci Med 62: 217–227. 16054281 10.1016/j.socscimed.2005.05.031

[16] Weir SS, Pailman C, Mahlalela X, Coetzee N, Meidany F, et al (2003) From people to places: focusing AIDS prevention efforts where it matters most. AIDS 17: 895–903. 12660537 10.1097/01.aids.0000050809.06065.e0

[17] Weir SS, Morroni C, Coetzee N, Spencer J, Boerma JT (2002) A pilot study of a rapid assessment method to identify places for AIDS prevention in Cape Town, South Africa. Sex Transm Infect 78: i106–113. 12083428 10.1136/sti.78.suppl_1.i106 PMC1765815

[18] Napper LE, Fisher DG, Reynolds GL, Johnson ME (2010) HIV risk behavior self-report reliability at different recall periods. AIDS Behav 14: 152–161. 19475504 10.1007/s10461-009-9575-5 PMC2814040

[19] Kalichman SC, Williams EA, Cherry C, Belcher L, Nachimson D (1998) Sexual coercion, domestic violence, and negotiating condom use among low-income African American women. J Womens Health 7: 371–378. 9580917 10.1089/jwh.1998.7.371

[20] Raiford JL, Diclemente RJ, Wingood GM (2009) Effects of fear of abuse and possible STI acquisition on the sexual behavior of young African American women. Am J Public Health 99: 1067–1071. 19372531 10.2105/AJPH.2007.131482 PMC2679788

[21] Mittal M, Senn T, Carey M. Fear of violence consequences and condom use among women attended a STD clinic. Under review. 10.1080/03630242.2013.847890 PMC3841968 24215273

